# The BREATH-TRACHER 2 Trial: Protocol for a Retrospective Mixed Methods Study to Establish the Utility of a Wearable Device in the Detection of Chronic Obstructive Pulmonary Disease Exacerbations

**DOI:** 10.2196/79503

**Published:** 2025-12-24

**Authors:** Beyza Toprak, Louise Hamilton, Burcu Kolukisa Birgec, Alexander Balfour Mullen

**Affiliations:** 1 Strathclyde Institute of Pharmacy and Biomedical Sciences, University of Strathclyde, 161 Cathedral Street, Glasgow, G4 0RE, United Kingdom, 44 1415484409, 44 1415522562; 2 Vale Centre for Health and Care, Glasgow, United Kingdom

**Keywords:** chronic obstructive pulmonary disease, respiratory, home monitoring, wearable device, respiration, vital signs, digital health, acute, feasibility study, protocol, COPD, exacerbation

## Abstract

**Background:**

Acute exacerbations of chronic obstructive pulmonary disease (eCOPD) are a major clinical challenge, often leading to frequent emergency room visits and significantly reducing patients’ quality of life. Early detection through wearable devices could facilitate timely interventions at the community level, reducing hospital admissions and disease-related morbidity and mortality.

**Objective:**

This study seeks to retrospectively assess the feasibility and reliability of the Frontier X2 wearable device for continuous monitoring of clinically relevant physiological signals in volunteers with chronic obstructive pulmonary disease (COPD) who experience acute exacerbations. In particular, the study aims to characterize early physiological alterations that may occur prior to the exacerbation of COPD, assessing the ability of the device to capture these changes in the real-world settings.

**Methods:**

This single-center feasibility study involves prospective physiological data collection with retrospective analytical evaluation of physiological changes occurring around clinically confirmed COPD exacerbations. A total of 30 participants with COPD (Modified Medical Research Council Grades 1‐4) who have experienced at least one exacerbation within the past 12 months will be included. Each participant will wear a continuous physiological monitoring device in their free-living environment for up to 18 months or until an eCOPD event occurs. The device will continuously collect physiological signals, including heart rate, respiratory rate, and heart rate variability. Data will be analyzed using statistical quality control methods, specifically the Cumulative Sum approach, to identify physiological deviations occurring within 7 days (168 h) prior to an exacerbation event. These findings will be correlated with clinical records and qualitative data. Qualitative information will be obtained through biweekly self-administered questionnaires assessing symptom changes and through additional adherence and usability questionnaires completed during home visits conducted every 2 weeks by the researcher.

**Results:**

Recruitment for this study started in June 2024 and was completed in July 2025. It is anticipated that data collection will be completed within 18 months of study initiation, with data analysis finalized by December 2025. Final results will be published in January 2026.

**Conclusions:**

The BREATH-TRACHER 2 study will evaluate the use of the Frontier X2 device in participants with COPD in home settings. The Frontier X2 device, if successful, has the potential to transform COPD management and support proactive care, leading to enhanced clinical outcomes and reduced disease mortality and morbidity.

## Introduction

### Background

Chronic obstructive pulmonary disease (COPD) represents a significant public health challenge, with individuals often enduring the disease for many years [1]. It ranks among the top three leading causes of death globally, responsible for 3.23 million deaths in 2019 [2], and the United Kingdom has the third highest COPD mortality rate in Europe [3]. Projections suggest that by 2060, annual global deaths attributable to COPD will exceed 7 million [4].

In the United Kingdom, the burden is equally concerning. In 2019, approximately 21,000 COPD-related deaths were reported [5], while more recent estimates indicate that COPD claims around 30,000 lives annually in the United Kingdom [6]. Currently, 1.4 million people in the United Kingdom are diagnosed with COPD, a figure that has risen steadily over the past decade [7] and is projected to be 1.62 million by 2040 [8]. Each year in the United Kingdom, around 115,000 people are newly diagnosed with COPD, equating to a diagnosis occurring every 5 minutes [3].

Globally, the World Health Organization estimates that 64 million people are living with COPD [9]. In the United Kingdom, the economic burden of the disease is of major concern. Annual management costs to the National Health Service (NHS) are estimated at £1.9 billion (US $3.13 billion) [10], and COPD accounts for 29% of all respiratory disease–related health care expenses, as shown in Table 1. Given that the total cost of respiratory diseases has been estimated at £165 billion (US $271.6 billion), COPD alone contributes approximately £48 billion (US $79 billion) to this burden [10].

**Table 1.** Economic burden of COPD and total lung conditions in the United Kingdom, based on 2014 estimated data^a^.

	Annual expenditure (£m)	
Category	COPD expenses	Total lung conditions
Direct costs	1847	9895
Indirect costs	61	1222
Intangible costs	46,636	154,241
Total economic burden in the United Kingdom	48,545	165,357

a Adapted from [10].

Emergency hospital admissions constitute the largest share of COPD-related costs, representing 72.8% of the total, while prescription medications account for 12.2% [11]. Exacerbations further intensify the economic impact, with moderate episodes costing approximately £118 and severe episodes ranging between £3,329 and £3,726 across UK territories [12,13].

The overall yearly direct health care expenses for individuals with COPD in England, which encompass the costs of moderate and severe exacerbations as well as maintenance, were projected to rise from £1.50 billion in 2011 to £2.32 billion by 2030 [12]. This data highlights the urgency for innovative health care models focused on early intervention and the prevention of exacerbations.

The clinical course of COPD is marked by chronic respiratory symptoms, airflow limitation resulting from airway or alveolar abnormalities, and exacerbations, which are pivotal events in disease progression [14]. These exacerbations, defined by the Global Initiative for Chronic Obstructive Lung Disease, involve an acute worsening of respiratory symptoms beyond typical day-to-day variation. They are often triggered by bacterial or viral infections, environmental factors like air pollution or weather changes, and occasionally by unknown factors [1]. Exacerbations significantly impact patient outcomes, including increased hospitalization rates, a decline in quality of life, and heightened mortality risks [15,16]. Moreover, exacerbations serve as indicators of disease progression and predictors of future health outcomes [17].

Unreported exacerbations remain a persistent challenge in COPD management, often influenced by factors such as age, gender, and socioeconomic status [18]. The failure to report these events leads to undertreatment, prolonged recovery, and diminished quality of life while also underestimating the patient’s risk of subsequent exacerbations. Treatment delays further exacerbate outcomes, with research indicating a median delay of 4.8 days from onset of an exacerbation to recognition, 9.3  days from recognition to action, and 8.3  days from recognition to general practitioner (GP) visit [19]. Early treatment is associated with faster recovery, reduced hospital admissions, and improved health-related quality of life, highlighting the importance of prompt medical intervention and patient education regarding exacerbation symptoms [20]. The management of acute exacerbations of COPD emphasizes the significance of effective disease management, which includes implementing a self-management plan that features a self-administered rescue pack containing antibiotics and steroids [21]. However, there is currently a limited understanding of patient self-management and appropriate use of the rescue pack. This often results in improper medication usage, delays in treatment, and instances of both overuse and underuse of medications [21,22].

Preventing hospital readmissions is a key strategy in alleviating the overall burden of COPD. Understanding the physiological changes that occur during exacerbations is essential for improving disease management. Studies show that some physiological signals begin to change before an overt clinical exacerbation, providing an opportunity for earlier detection and intervention [23,24]. Continuous monitoring of vital signs also has the potential to reduce the frequency and severity of exacerbations. However, earlier studies have encountered limitations such as small participant samples, potential conflicts of interest, and the complexity of monitoring devices that require frequent and precise usage [25]. Although advancements in wearable technologies have improved accessibility for continuous vital sign monitoring, their accuracy and reliability remain a cause for concern [26].

The BREATH-TRACHER 2 study aims to utilize the Frontier X2 wearable device to detect early indications of exacerbations of COPD (eCOPD). The aim is to identify physiological changes sooner than otherwise feasible, which could help reduce hospitalizations and alleviate the overall burden of the disease. By monitoring heart rate (HR), breathing rate (BR), and other physiological parameters in individuals with COPD, this study aims to assess whether early signal changes can be detected prior to a clinical exacerbation. Earlier identification may support timelier treatment and could potentially reduce the impact of exacerbations in future applications.

In the literature, several studies have investigated the use of wearable technologies to detect eCOPD using physiological parameters such as HR, BR, and HR variability (HRV). These parameters have shown promising potential as early indicators of eCOPD. However, most previous studies have relied on photoplethysmography or accelerometer-based technologies, which are more susceptible to motion artifacts, environmental noise, and signal interruptions—particularly in free-living conditions. While the goal of these studies was to enable remote, real-world monitoring, the accuracy of photoplethysmography and motion-based sensors can be significantly affected by such external factors.

Remote monitoring technologies are increasingly used for the diagnosis, treatment, and tracking of disease progression across a wide range of conditions. They can be evaluated and compared according to factors such as predictive accuracy, cost, usability, patient comfort, and physical design [26]. Some earlier studies have employed bulky or portable systems that are unsuitable for continuous monitoring in daily life, whereas lightweight, user-friendly devices are better suited for long-term free-living applications and can improve adherence and data quality. Device cost is also a key consideration for large-scale clinical deployment.

The Frontier X2 device used in this study operates using continuous single-lead electrocardiography (ECG) technology, which measures the heart’s electrical activity through electrodes embedded in a chest strap. The device records the depolarization and repolarization of cardiac muscle during each heartbeat, producing high-fidelity HR and BR signals derived directly from the ECG waveform. The system automatically filters noise from muscle movement and other artifacts, ensuring stable data capture. Frontier’s proprietary software detects R-peaks and provides raw respiratory rate (RR) intervals, from which HRV parameters such as the SD of normal-to-normal intervals can be calculated. Owing to its lightweight design (approximately 20 g), continuous data collection capability, and relatively low cost, the Frontier X2 represents a practical and reliable option for long-term physiological monitoring in COPD populations.

### Rationale for Study and Potential Impact

The rationale of the study is to address the need for earlier identification of an acute exacerbation in people with COPD and to explore the potential of remote monitoring using a Frontier X2 wearable device to monitor changes in RR and cardiac signals (HR and HRV).

Detecting exacerbations at an early stage is crucial as timely intervention and appropriate management can prevent severe exacerbations and their associated complications. By utilizing a wearable device for remote monitoring, the study aims to continuously collect physiological signals from patients with COPD during their daily activities. This approach offers the opportunity to monitor patients in their natural environment and capture subtle changes that may indicate disease progression or exacerbations before they are clinically conspicuous.

### Aims and Objectives

#### Aims

The study aims to establish whether the Frontier X2 wearable device is suitable to monitor the clinical health of a person with COPD and immediately identify when it begins to deteriorate. Earlier notification of disease worsening could improve rate of patients seeking therapy at a community level and reduce likelihood of hospital admission. Additionally, a wearable device could provide real-world insights into the effectiveness of regular prescribed medications and measure the impact of any rescue interventions.

#### Primary Objective

The primary purpose of the study is to retrospectively establish if the Frontier X2 medical device can accurately identify when a person with COPD is starting to experience a clinical exacerbation.

#### Secondary Objectives

The secondary objectives of this study are to (1) explore if the device can monitor volunteer compliance of their inhaler usage; (2) explore instances of signal-clinical discordance (ie, situations where physiological patterns captured by the device do not fully align with the clinically documented eCOPD event); (3) explore if the device can stratify disease severity in individual volunteers; (4) explore if the device can identify responsiveness to intervention treatment following a COPD exacerbation; (5) assess device adherence, usability, and comfort;, and (6) explore how soon the device can identify when a person with COPD is starting to experience a clinical exacerbation.

## Methods

### Overview

An eCOPD refers to an acute worsening of dyspnea, cough, and sputum production occurring within a 14- day period. It may also present with tachypnea and tachycardia and is commonly associated with local or systemic inflammatory responses triggered by respiratory infections, environmental pollutants, or other irritants affecting the airways [1]. eCOPD is considered a sign of disease progression, a predictor of future health and outcomes, and a leading cause of morbidity and mortality, and it increases disease costs [17]. Early identification and timely reporting of symptom deterioration are therefore essential to minimize hospitalization and reduce disease burden, underscoring the importance of reliable exacerbation detection in patients with COPD.

Building on this rationale, this study adopts a mixed methods, observational, open-label, uncontrolled cohort design with prospective physiological data collection and retrospective analytical evaluation to explore the feasibility and potential of continuous physiological monitoring for detecting early changes of eCOPD. Participants will be continuously monitored throughout their participation in the study, including before, during, and after their COPD exacerbation using a wearable device that records physiological signals. The 168-hour pre-exacerbation period and the subsequent return-to-baseline period after the event will be retrospectively examined using the physiological data captured by the device. COPD exacerbation will be identified and cross-validated from information provided by participants via individual COPD symptom questionnaires and cross-validated through clinical records in the participants’ GP electronic medical system. The study aims to recruit 30 volunteers who have previously experienced COPD exacerbation-induced hospitalization within a 1-year period prior to recruitment.

There is no specific medical intervention planned as part of the study; participants will continue to receive their usual standard of care throughout the study period. However, wearing a medical monitoring device may lead to behavioral changes (Hawthorne effect) among participants, such as increased disease awareness or improved adherence to medication (eg, inhaler) use. These potential behavioral effects are acknowledged as possible outcomes of the monitoring process.

### Primary and Secondary End Points

#### Primary End Point

The primary end point is the retrospective analysis of physiological signals following a clinically validated COPD exacerbation. Exacerbations will be confirmed via the electronic GP system, either upon hospital admission or when managed at home using a prescribed rescue pack.

We will retrospectively analyze physiological data to identify the earliest point at which alterations in signals become detectable prior to a clinically confirmed exacerbation. The analysis will continue until the participant’s signals return to baseline. This window is expected to span approximately 7 days (168 h); both the timing and duration of physiological deviations may vary depending on device sensitivity and the nature or severity of the exacerbation, and these variations will be examined descriptively. This study aims to investigate these pre-exacerbation physiological alterations and evaluate the potential of the wearable device to detect these early changes, potentially supporting future clinical monitoring and management in individuals with COPD.

#### Secondary End Points

The secondary end points are to (1) evaluate the everyday usability of the wearable device in a primary care setting; (2) appraise the ability of the wearable device to monitor daily disease burden and impact of regular medication use on the long-term health management of a person with COPD; (3) determine the potential of the wearable device to assess the effectiveness of any clinical intervention provided during an exacerbation.

### Study Design

#### Recruitment

**Figure 1.**
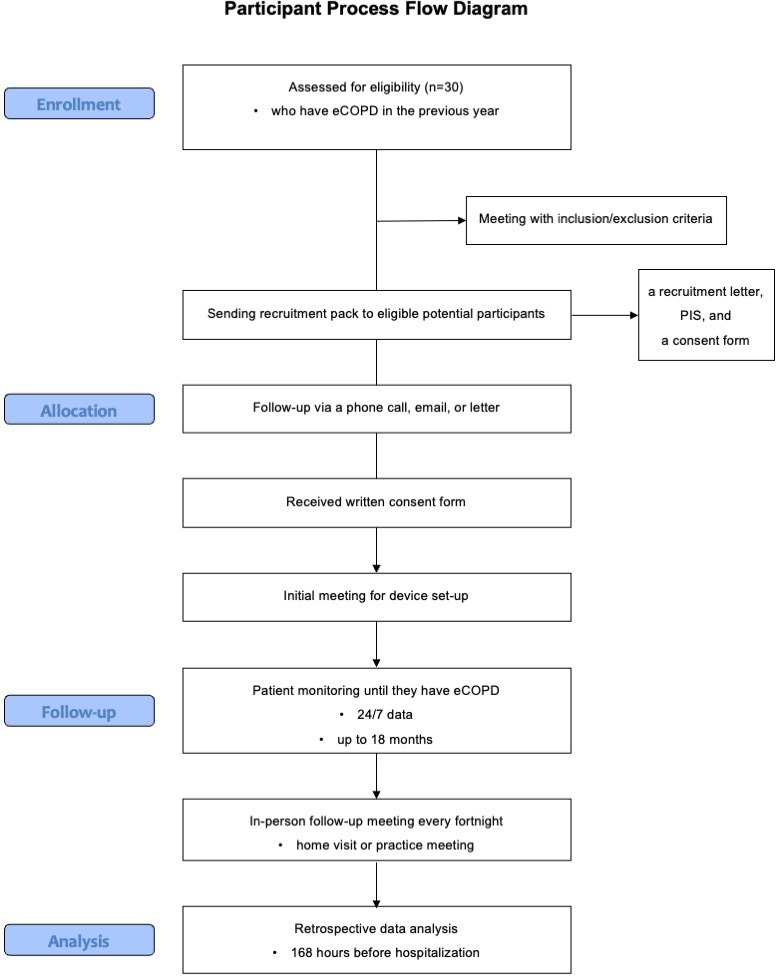
Participant process flow diagram.

The SPIRIT guidelines were used in the construction of this manuscript (Checklist 1)*.* The participant process flow diagram (Figure 1) outlines the study process. Eligible volunteers will be recruited through NHS Scotland’s Primary Care clinical database. Those who qualify will receive a recruitment pack that contains a recruitment letter, participant information sheet, and consent form and will have 2 weeks to review the materials, discuss the study with an independent researcher, and decide whether they wish to participate. Following this period, a follow-up telephone call will be made to confirm the receipt of the recruitment pack and to determine if the individual is interested in joining the study.

Individuals expressing interest will be asked to provide signed consent and will be invited to a screening visit, which can take place either at the surgery or in their home. During this visit, the study will be explained in detail, and any additional questions will be addressed. Participants will have the opportunity to view and test the wearable device, as well as learn how to use the accompanying app, with support from the research team to set up the device and initiate their participation in the study.

Participants will have the freedom to withdraw from the study at any time and for any reason without any impact on their care and without the obligation to provide a reason for their withdrawal.

#### Participant Training

Each participant will be provided with the necessary equipment, including the Frontier X2 device, battery charging materials, cleaning products, and a detailed instruction manual. Participants will also be required to download the Frontier X2 app onto a suitable smartphone, and they will be creating an account using their designated study code and email. For participants who do not own a smartphone, one will be supplied by the research team. Similarly, those without a stable Wi-Fi connection will receive a SIM card to enable cellular data access.

Throughout the participation period, participants will receive guidance on how to properly use and maintain the device, which includes instructions on the following:

- Device usage: How to wear and operate the Frontier X2.
- Data collection: Procedures for syncing data with the app.
- Charging protocols: Ensuring the device remains functional throughout the study.
- Light signals: Understanding the various light patterns emitted by the device, which indicate its status.

#### Patient Population Size

The study will recruit a total of 30 volunteers, all of whom have been diagnosed with COPD and have experienced at least one exacerbation, resulting in hospitalization within the 12 months prior to enrollment. This inclusion criterion increases the likelihood that participants will experience an additional exacerbation during the 18-month follow-up period.

According to a previous study [27], it has been found that COPD exacerbation rates range from 0.5 to 3 events per person per year. Over an 18-month period, this corresponds to an expected 0.75 to 4.5 exacerbations per individual. While only the first exacerbation is relevant to the primary end point of this study, this range suggests that a large proportion of participants—potentially 70% or more—are likely to experience at least one exacerbation within the study timeframe. Therefore, a sample size of 30 participants is expected to yield a sufficient number of exacerbation events for retrospective physiological signal analysis, while accounting for variability in exacerbation frequency and potential loss to follow-up.

#### Study Assessments

A researcher will separately meet each participant every 2 weeks to monitor progress and enquire whether they have experienced any exacerbations that have required medical attention. Two questionnaires will be completed by the participant. A COPD symptom questionnaire is used to assess any changes in the participant’s symptoms as independent variables, including appetite, breathing, cough, energy levels, general wellness, and medication usage. In addition, a Device Usage and Adherence Questionnaire will evaluate the participant’s experience with the Frontier X2 device, focusing on ease of use, adherence to the study protocols, and any challenges they may have faced while using the device. Following the completion of the questionnaires, the researcher will address any technical issues related to the device or app, offer troubleshooting assistance, and answer any questions the participant may have about the study. These visits will ensure regular communication and allow the researcher to provide support, ensuring that participants are comfortable with the device and that any concerns are promptly resolved.

### Study Setting

The primary setting for this study will be remote, real-time home-based monitoring, allowing participants to use the device in their everyday environment. Data will be collected continuously in this real-life setting, ensuring that the study reflects the participants’ typical routines and lifestyle conditions, thereby enhancing the validity of the results.

### Study Participants

#### Inclusion Criteria

The participant must meet all the following criteria to be considered eligible for the study: (1) any person aged 18 years or over, (2) current diagnosis of patients with COPD who have experienced exacerbation-induced hospitalization in the previous year, (3) be willing and able to comply with study procedures and be available for study visits, (4) be able to use a “smartphone, tablet, or computer,” (5) be able to give written consent, and (6) able to understand written and spoken English.

#### Exclusion Criteria

The participant may not enter the study if any of the following applies: (1) the inability to give written informed consent; (2) known respiratory disorders other than COPD which, in the opinion of the investigator, is the main contributor to the patient’s symptoms (eg, asthma, lung cancer, sarcoidosis, other interstitial lung diseases, tuberculosis, lung fibrosis, cystic fibrosis, and non-COPD-related bronchiectasis); (3) a known history of significant systemic and other organ-related diseases, other than COPD, which, in the opinion of the investigator, is likely to interfere with the study or impact on participant safety (eg, severe rheumatoid arthritis and lupus, kidney, liver, endocrine, psychological disorders); (4) known to be severely alpha-1-antitrypsin deficient (PiSZ or PiZZ); (5) any known history of violence/chaotic substance misuse based on their medical records; (6) having undergone lung surgery (eg, lung volume reduction, lobectomy) within the last 6 months; (7) have cancer or other terminal condition which, in the opinion of the investigator, has a mortality of 12 months or less; (8) taking high-dose oral corticosteroid medication (equivalent to a daily dose of ≥10 mg of prednisolone) for more than 3 consecutive months; (9) being pregnant; (10) patients already involved in an ongoing research study; (11) participation in an interventional clinical study within 3 months of Visit 1 or participation in a study using an investigational medicinal product either in the previous 3 months or in the interval from last using the investigational medicinal product to 5 times its half-life; (12) patients with other significant lung disease, unable to consent, unable to use the technology (eg, inability to use the data device or complete the questionnaires), or at the clinician’s discretion for other more significant medical/social reasons; (13) known allergy to any chest strap; (14) on long-term oxygen therapy; and (15) acute exacerbation of COPD within 6 weeks prior to inclusion.

### Study Device

The Frontier X2 is a chest-worn wearable device designed to monitor physiological parameters in real time. This compact device captures key metrics including HR, BR, HRV, continuous ECG, and physical activity, making it well-suited for continuous health monitoring. The device employs Bluetooth Low Energy technology for data synchronization and is IP67-rated, providing water resistance up to 1.5 m. It also meets international industry standards including International Electrotechnical Commission; International Organization for Standardization; Registration, Evaluation, Authorization and Restriction of Chemicals; Conformité Européenne; and Restriction of Hazardous Substances, ensuring regulatory compliance and durability.

Although initially developed for sports performance monitoring, the Frontier X2 incorporates features with strong relevance for clinical research, such as extended ECG recording and onboard vibration alerts. In this study, the device will be used in passive data collection mode, without real-time alerts being activated. Data are stored locally for up to 24 hours. Given the protocol requirements, participants will be asked to sync data daily via Bluetooth.

Previous studies have demonstrated that the Frontier device has the potential for disease management for people with different chronic diseases [28,29]. Ongoing and future research, including the present study, aims to contribute to the growing body of evidence regarding its accuracy, reliability, and potential applications in chronic disease management. Full specifications and an image of the Frontier X2 can be found at the manufacturer’s website [30].

### Monitoring

The study will monitor both subjective symptom scores and objective physiological metrics to evaluate participants’ health status. Symptom scores will include measures related to appetite, breathing, cough, energy levels, overall wellness, and medication usage, providing insights into fluctuations in COPD symptoms (see Multimedia Appendix 1). Objective physiological parameters, such as HR, BR, ECG, HRV, strain, and daily step count, will be continuously recorded to identify significant changes potentially associated with COPD exacerbations.

Physiological data are collected prospectively, but no real-time clinical monitoring or automated alerts will be used. Data will be reviewed only during scheduled biweekly visits, and participants initiated contact if they reported worsening symptoms, device issues, or unexpected physiological readings. This ensures that the study remains observational and noninterventional. No real-time clinical monitoring or medical decision-making will be performed.

This combined approach, integrating subjective symptom scores with objective physiological data, aims to provide a comprehensive analysis of factors contributing to COPD exacerbations, enabling timely interventions and improved disease management.

### Data Collection

Biometric data will be continuously uploaded to a secure cloud platform, ensuring that the integrity and fidelity of the data are always maintained. The biometric data will be automatically synchronized with the cloud system via the Frontier X2 device and its associated application. In the event of an automatic synchronization failure, participants will be instructed to manually sync the data within the app, thereby ensuring that all relevant information is captured. Wear-time expectations are not enforced; instead, actual wear-time (days/hours worn) will be recorded and reported as an adherence outcome.

The analysis of the biometric data will be conducted using proprietary software developed by the device manufacturer, which is designed to prevent any manipulation that could introduce bias into the analytical process. This rigorous approach to data collection safeguards the validity of the findings and upholds the principles of good clinical practice.

Qualitative data will be collected through a self-administered questionnaire (see Multimedia Appendix 2). This qualitative information will be transcribed into an electronic file, and data integrity will be verified by an independent third-party researcher.

Statistical analyses performed on the collected data will undergo scrutiny by an independent University statistician. No observational data will be collected during this study.

At the conclusion of the project, patients will be provided with a lay summary of the research findings, and it will be additionally distributed through a variety of channels, such as the University website, media outlets, and charities, such as the British Lung Foundation and Chest Heart & Stroke Scotland.

### Data Analysis

The data analysis will incorporate both qualitative and quantitative methodologies to ensure a thorough evaluation of the collected data.

#### Qualitative Analysis

Thematic analysis will be undertaken to identify and interpret key themes, categories, and patterns emerging from the qualitative data. Using NVivo software, coding and categorization will facilitate a structured and systematic approach to data interpretation, enabling researchers to gain deeper insights into participants’ experiences and perceptions. This process will provide a foundation for generating hypotheses and addressing the study’s research objectives.

#### Quantitative Analysis

Time-series analysis will be conducted on physiological data to identify patterns and trends over time. Statistical software including SPSS, Minitab, and Python will be used for data processing and analysis. Control chart techniques, particularly the Cumulative Sum (CUSUM) chart, will be applied to evaluate physiological changes before, during, and after COPD exacerbations. The adequacy of the selected time-series models will be assessed through autocorrelation analysis and examination of residual distributions to ensure accurate representation of the underlying data structure.

Physiological parameters, including HR, HRV, and its derived metrics—the root mean square of successive differences, the SD of normal-to-normal intervals, low-frequency power, high-frequency power, and the low-frequency to high-frequency ratio—as well as RR, will be used for quantitative analysis. As this feasibility study is conducted in free-living conditions, it is not possible to fully control contextual factors that may influence HR, RR, or HRV, such as medication changes, intercurrent infections, sleep variability, or changes in daily activity. These factors will therefore be documented during biweekly follow-up visits and reviewed descriptively when interpreting physiological deviations. Analyses will focus on within-participant deviations from each individual’s baseline rather than between-participant comparisons.

The CUSUM chart approach will follow methodologies previously described by Montgomery [31], which enables detection of small, sustained deviations from baseline values in time-series data. For each participant, baseline data will correspond to a stable period with no reported symptom worsening—specifically, the first 7 days of participation. The reference value (k) and decision threshold (h) will be individually calculated based on this baseline period. In accordance with the inclusion criteria, all participants must have been free from any eCOPD events for at least 6 weeks prior to recruitment, supporting the selection of the initial stable days as an appropriate baseline.

CUSUM chart performance will be evaluated in terms of sensitivity and specificity. Sensitivity will be defined as the correct detection of a true eCOPD event when the cumulative deviation exceeds the decision threshold (h) within the defined pre-event window. Specificity will be defined as the absence of false alarms (ie, no threshold exceedance) during stable periods. Validation will be performed at both individual and group levels to estimate participant-specific and overall sensitivity and specificity.

The pre-event window will be defined as 7 days (168 h) prior to the clinically confirmed onset of exacerbation; however, this duration may vary among individuals depending on their clinical course. Some participants may require hospital admission on the first day of symptom worsening, whereas others may experience a delay of several days. While all available data will be analyzed, the primary focus will remain on the 7-day pre-exacerbation period.

Missing data handling will involve evaluating several imputation techniques, including interpolation, Kalman filtering, Last Observation Carried Forward, and Predictive Mean Matching. The most appropriate method will be selected for each dataset based on data structure and continuity.

If a participant experiences more than one eCOPD event during the study period, each episode will be treated as a separate exacerbation. To minimize participant burden, data collection will be terminated immediately after confirmation of an eCOPD event through the participant’s GP record. CUSUM-detected signals will be cross-validated against contemporaneous meeting notes obtained during regular follow-up sessions to ensure consistency with the first self-reported symptoms of worsening.

The onset time of each COPD exacerbation will be confirmed using clinical information from GP and hospital records. The onset will be defined as either the date on which the participant begins using their prescribed rescue pack or the date of hospital admission.

In this study, the aim was to observe and analyze each participant’s adherence and device usage in their natural living environment without imposing any burden or obligation to use the device. Adherence, device usage, and the duration of recorded data will be reported as study outcomes to evaluate the practicality and real-world applicability of using a wearable device in individuals with COPD. Since one of the main challenges of wearable or medical devices in free-living conditions is maintaining consistent adherence, no fixed adherence target was predefined. Instead, the number of days and hours the device was worn will be reported as adherence outcomes. This approach enables assessment of device acceptability, user compliance, and usage patterns, particularly during eCOPD periods, during unsupervised, real-world conditions without external reminders or enforced wearing schedules.

### Ethical Considerations

This study complies with the Declaration of Helsinki [32] and adheres to Good Clinical Practice guidelines [33]. Participation in the study is completely voluntary, and participants may withdraw at any time without providing a reason, with no impact on their future medical care. Ethical approval for the study was granted by the NHS London - Stanmore Research Ethics Committee (Protocol number UEC23/71; IRAS project ID 32,823), and the study has been registered on ClinicalTrials.gov (NCT06419062). If there are any significant deviations from the study protocol, the investigator will submit an amendment for further approval by the ethics committee. No participants were compensated during their involvement in the study.

### Data Management

All data collected in this project will comply with the General Data Protection Regulation, the Caldicott Guardian principles, and the NHS Code of Confidentiality. Personal health data will be anonymized or pseudonymized wherever feasible and stored securely using encrypted electronic platforms. Short questionnaires completed by volunteers will be anonymized with unique numerical codes, and data will be transcribed into encrypted files. Researchers will adhere to the confidentiality policies of the University of Strathclyde and the UK General Pharmaceutical Council.

Anonymized physiological data will be securely stored within the Fourth Frontier Technologies Ltd cloud system, in full compliance with UK data protection and general data protection regulations. Data analyzed or generated at the University of Strathclyde will be securely stored within the university’s encrypted cloud infrastructure.

Each participant will be assigned a unique project identity code to ensure anonymity. The mapping file linking participant identity codes to personal information will be stored electronically in an encrypted cloud folder, with a physical backup kept in a locked filing cabinet at a secure university location. Access to these files will be strictly limited to the PhD researcher and their academic supervisor.

Hard-copy questionnaires completed by participants will be stored in a lockable briefcase until transferred to a secure university facility. Within 10 working days, responses will be transcribed into an encrypted digital file on the university cloud system, after which the physical copies will be shredded and disposed of through a university-approved confidential document disposal service.

To maintain confidentiality, all personal information will be pseudonymized, ensuring that no individual can be directly identified in the unlikely event of data loss. A unique email address and study ID will be generated for each participant to further protect identity. Within the Frontier dashboard, all uploaded physiological data are anonymized and accessible only via these identifiers.

For data processing and analysis, physiological recordings will be downloaded as CSV files from the Frontier dashboard for each activity session. All analyses will be performed independently of the manufacturer’s proprietary platform using open-source analytical tools such as Excel, Minitab, and Python. The Frontier device automatically uploads raw physiological data directly to the secure cloud without alteration. Although raw data are initially stored on the Frontier cloud, copies of all exported files will be securely downloaded and stored in the University of Strathclyde’s OneDrive system for independent analysis.

The University of Strathclyde will act as the data controller, and Fourth Frontier Technologies Ltd will act solely as the data processor, providing secure data transmission and temporary cloud storage during participant involvement.

## Results

Following ethical approval of the study protocol, the first patient was enrolled in June 2024, and recruitment was completed in July 2025. Data collection is ongoing and is expected to be completed by December 2025, within the planned 18-month study period. Statistical analysis is being conducted simultaneously with data collection. Final results are expected to be submitted for publication in January 2026. The findings, regardless of whether they are positive or negative, will be disseminated through peer-reviewed journals.

## Discussion

### Strengths and Limitations

There are no direct potential benefits to the patients while they participate since it is only an observational study. However, the research team will periodically review participants’ physiological data as part of the ongoing analysis. If the biometric data indicate a potentially concerning clinical pattern, the participant’s health care provider will be informed, which may prompt an unscheduled but potentially beneficial clinical intervention. There is also the possibility that agreeing to participate in the study may result in a “Hawthorne effect,” where there is a change in behavior as a response to observation and assessment either by the volunteer themselves (eg, better medication adherence, cessation of cigarette smoking) or by their health care provider (eg, more frequent monitoring or review of their medication).

The size of the cohorts and the duration of the study might not be enough to get accurate and expected results, but this is typical for most pilot studies, as we intend to determine the correct size and duration of the study required for expected results, which can be further used to plan future longitudinal studies. This can be partly mitigated by attempting to recruit people who are at high risk of exacerbation.

Previous studies have demonstrated that physiological changes, particularly increases in HR and RR, can occur up to 72 hours before an eCOPD event, supporting the feasibility of detecting early physiological alterations. However, these studies did not characterize these changes as continuous signal trends or apply them for the prediction of exacerbation onset. The present study aims to address this gap by identifying early physiological deviations that may serve as negative predictive signals and by exploring how these could inform the timely initiation of therapeutic interventions. Furthermore, by monitoring when physiological parameters return to baseline, this study has the potential to evaluate the effectiveness of treatment and the dynamics of recovery following an eCOPD event.

### Potential Challenges and Alternatives

The potential risks to participants are considered to be low or mild. Volunteers will have to continuously wear the device (Frontier X2). This may be associated with minor discomfort of the device being situated on their chest that may cause mild positional discomfort while undertaking certain activities, such as having to adopt an alternative sleeping position. The device is secured to the chest by an elastic chest strap, composed of a hypoallergenic material, although there is still a small possibility that it may cause a skin irritation if it is worn for prolonged periods in the same position.

The device has a battery life of 24 hours and can be fully recharged in 45 minutes. Users will be provided with the necessary equipment for charging the device and are expected to charge their device every 24 hours. This can be a burden for participants. Volunteers will be expected to complete a 10-minute symptom questionnaire every 2 weeks to see if this correlates with device biometric data capture. This questionnaire will be collected when the PhD researcher undertakes a site visit to the volunteer’s house to check the battery for the device and to provide any additional technical support. These would discontinue if there were any discomfort.

The non-invasive physiological measurements involve minimal burden.

### Conclusions

This study aims to build upon the limited existing validation of the Frontier X2 device for monitoring patients with COPD in real-life settings. The continuous monitoring capabilities of the device may support earlier clinical interventions, reducing the need for hospital admissions and improving patient outcomes. By shifting the focus from reactive to proactive care, such technologies have the potential to enhance clinical management and reduce morbidity and mortality rates associated with COPD.

## Supplementary material

10.2196/79503 Multimedia Appendix 1 Symptom and self-management checklist used during in-person follow-up visits. The checklist includes yes/no items on breathlessness, sputum production, cough, fatigue, appetite, medication adherence, hospital admissions, and self-management practices, as well as questions on recent symptom worsening or improvement.

10.2196/79503 Multimedia Appendix 2 Device-related questionnaire used to assess long-term usability and comfort of the wearable device.

10.2196/79503 Checklist 1 SPIRIT checklist.

## Abbreviations

BR: breathing rate
COPD: chronic obstructive pulmonary disease
CUSUM: Cumulative Sum
ECG: electrocardiography
eCOPD: exacerbation of chronic obstructive pulmonary disease
GP: general practitioner
HR: heart rate
HRV: heart rate variability
NHS: National Health Service
RR: respiratory rate
