# Supplementary material for: The BREATH-TRACHER 2 Trial: Protocol for a Retrospective Mixed Methods Study to Establish the Utility of a Wearable Device in the Detection of Chronic Obstructive Pulmonary Disease Exacerbations
Source: JMIR Res Protoc. 2025 Dec 24;14:e79503. doi: 10.2196/79503 (PMC12736667; doi:10.2196/79503)
Supplement: Multimedia Appendix 1 [file resprot-v14-e79503-s001.docx]

**COPD Checklist**

| **In-person Follow-up**  Participant Code: | | | | | | |
| --- | --- | --- | --- | --- | --- | --- |
| **Sex:** □Female □Male | | | | | **Age:** | |
| Diagnosis: | | | | | mMRC Grade:  GOLD classification: | |
| 1. **BASELINE SYMPTOMS –** Breathlessness on a regular day: mMRC ../4  - Daily sputum production: □ yes □ no - Colour: - Regular cough: □ yes □ no | | | | | | |
| **Recent change in symptoms** □ yes □ no  If yes, since when:  □ Sputum color:  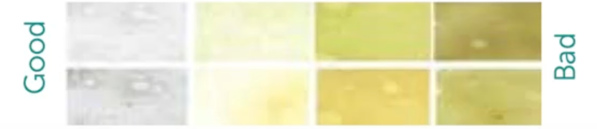  □ Dyspnea ↑ = ↓  □ Cough ↑ = ↓  □ Signs of hypercapnia  □ Sputum volume ↑ = ↓  □ Fatigue ↑ = ↓  □ Appetite ↑ = ↓  □ Energy/tiredness ↑ = ↓  □ Other  **CAT: /40** | | | | Maintenance medication/adherence/issues:  □ SABA □ LABA/LAMA  □ LABA □ LABA/ICS  □ LAMA □ ICS/LABA/LAMA  □Other:  Additional related medicines:  Non pharmacological Rx:  O_2_: CPAP: BIPAP: | | |
| 1. **RECENT ADMISSIONS AND EMERGENCY VISITS** | | | | | | |
| Hospital/ER | Where | Date | Length | | Reason (Dx) | Comments: |
| 1. **COPD Self-management (healthy behaviours) – Integrated (patient has used it in his/her daily life)?**   - Smoke-free environment □ yes □ no □ cannot tell  - Medication adherence □ yes □ no □ cannot tell  - Prevention/management of exacerbations □ yes □ no □ cannot tell  - Breathing control □ yes □ no □ cannot tell  - Stress management □ yes □ no □ cannot tell  - Physical activity and exercise □ yes □ no □ cannot tell  - Other _____________ □ yes □ no □ cannot tell  Comments and what patient should prioritize based on his/her need: | | | | | | |
| - **Participatory battery check:** □ yes □ no - **Date:** | | | | | | |
| Any other information communicated by volunteer during visit: | | | | | | |
