# Supplementary material for: The BREATH-TRACHER 2 Trial: Protocol for a Retrospective Mixed Methods Study to Establish the Utility of a Wearable Device in the Detection of Chronic Obstructive Pulmonary Disease Exacerbations
Source: JMIR Res Protoc. 2025 Dec 24;14:e79503. doi: 10.2196/79503 (PMC12736667; doi:10.2196/79503)
Supplement: Multimedia Appendix 2 [file resprot-v14-e79503-s002.docx]

**Participant Survey**

The Use of Medical Devices to Monitor COPD Patients

Breath Tracher 2 Study

Please circle your answer.


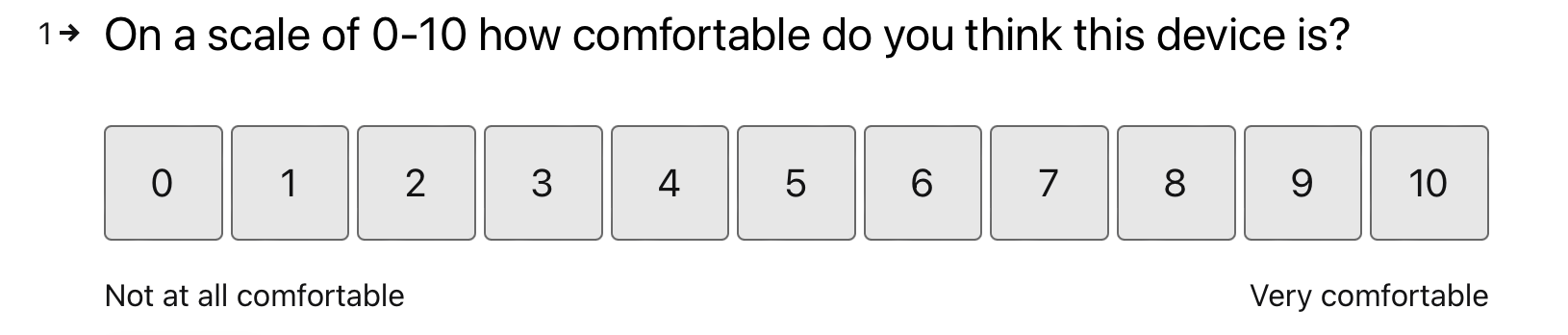


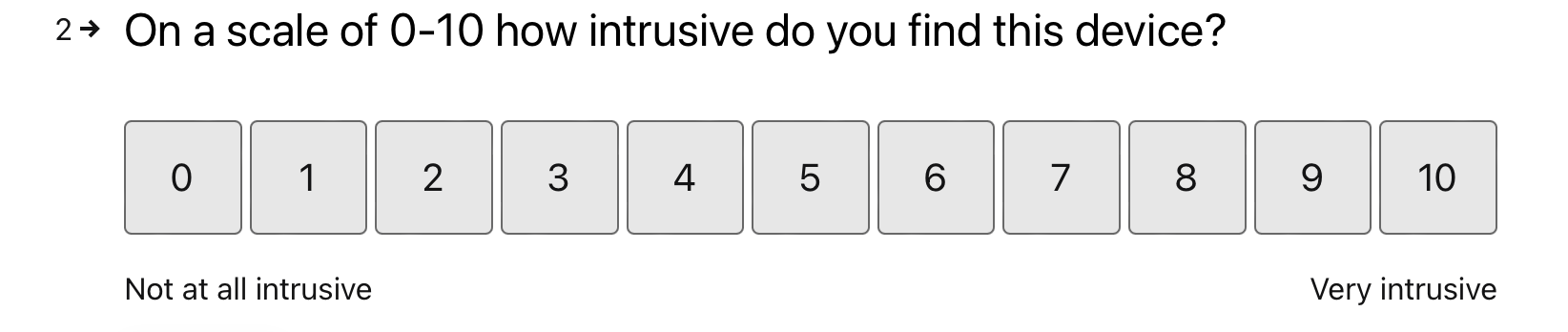


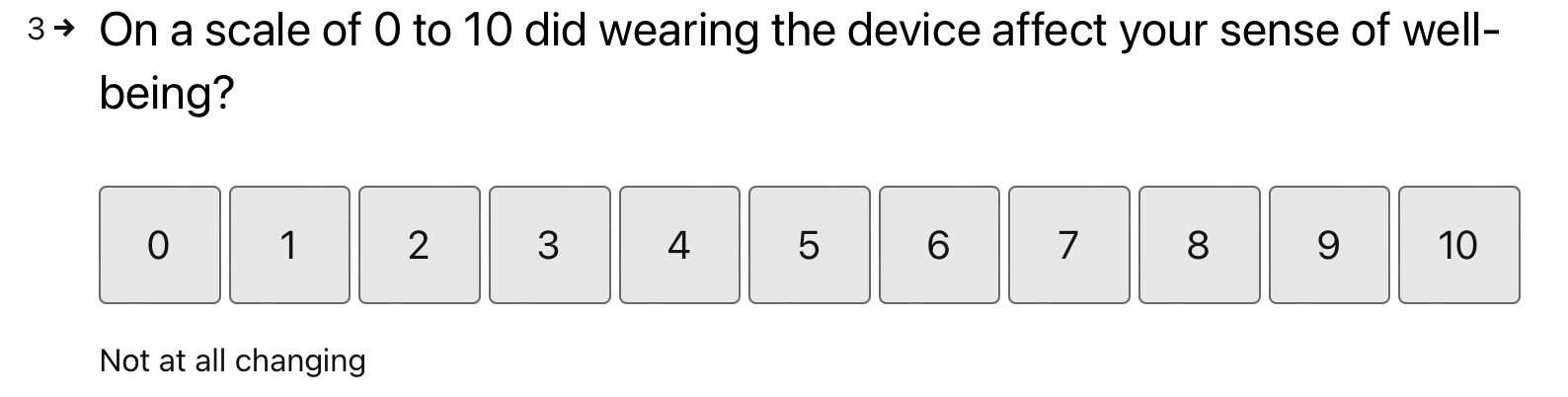


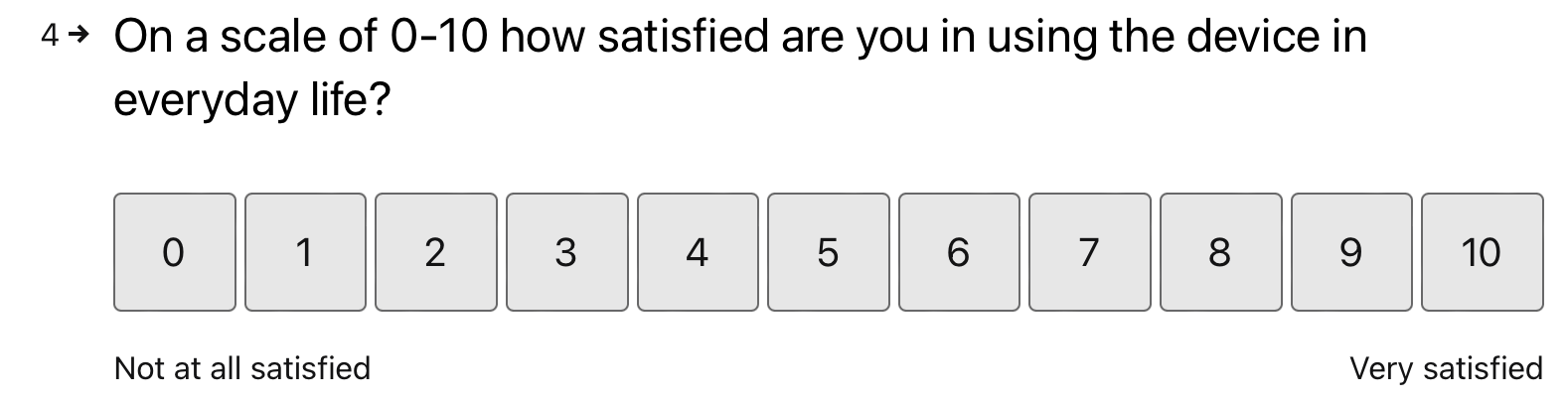


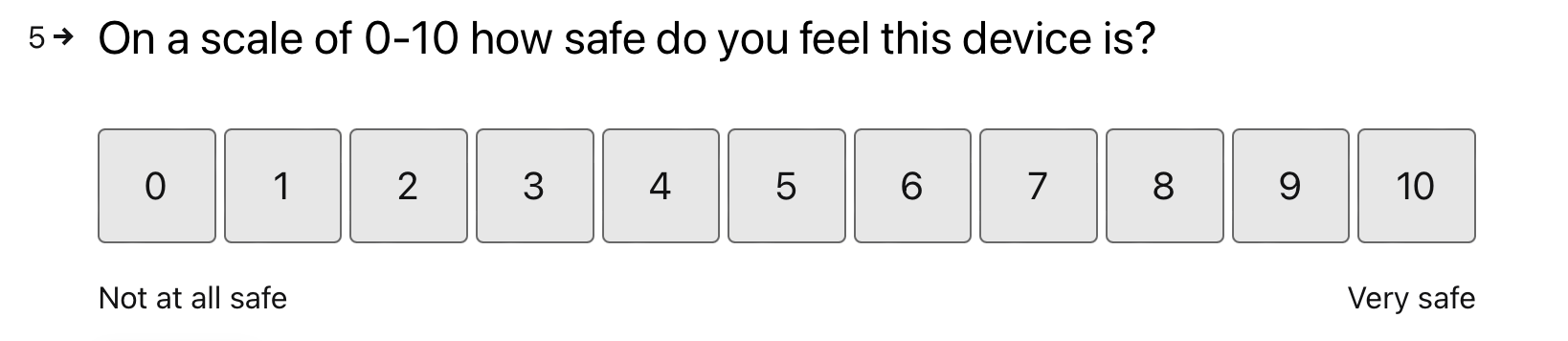


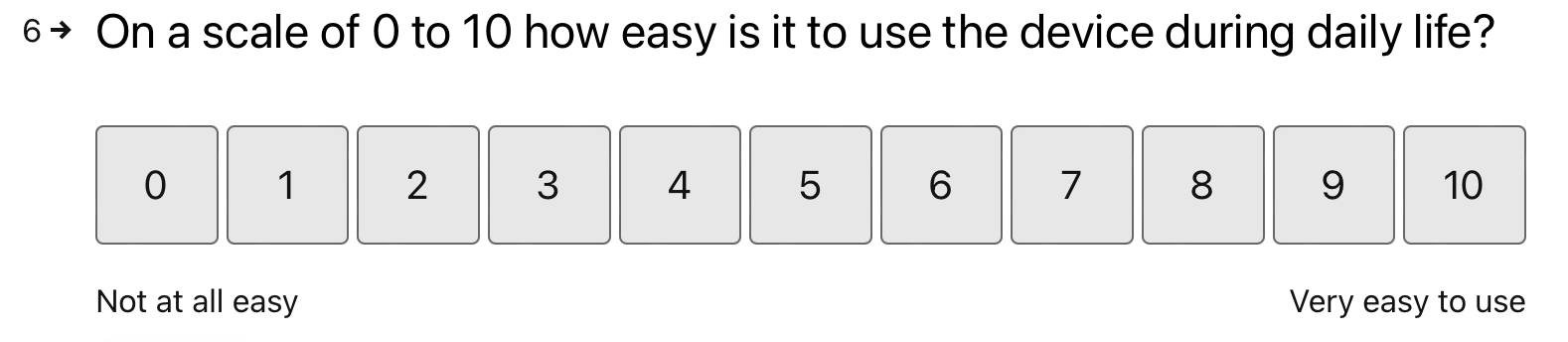


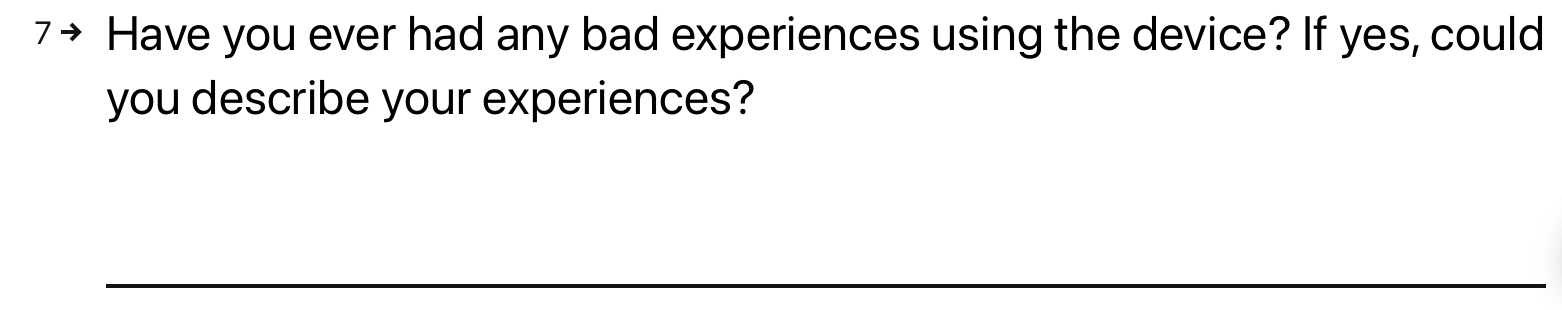


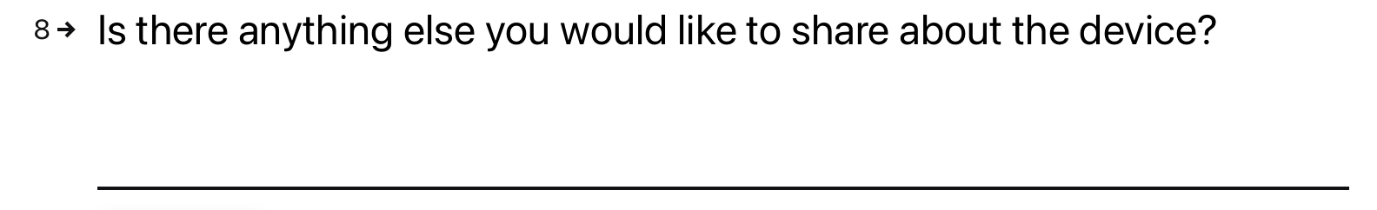


Participant Code: Researcher Name:

Initials: Sign:
